# Nrf2 in the Field of Dentistry with Special Attention to NLRP3

**DOI:** 10.3390/antiox11010149

**Published:** 2022-01-12

**Authors:** Lisa Schieffer, Claudia Manzl, Christoph Schatz, Johannes Haybaeck, Adriano Crismani

**Affiliations:** 1 Department of Dental and Oral Medicine and Cranio-Maxillofacial and Oral Surgery, Medical University of Innsbruck, MZA, Anichstr. 35, A-6020 Innsbruck, Austria; lisa.schieffer@i-med.ac.at; 2 Institute of Pathology, Neuropathology and Molecular Pathology, Medical University of Innsbruck, A-6020 Innsbruck, Austria; claudia.manzl@i-med.ac.at (C.M.); christoph.schatz@i-med.ac.at (C.S.); 3 Diagnostic & Research Center for Molecular BioMedicine, Institute of Pathology, Medical University of Graz, A-8036 Graz, Austria

**Keywords:** NLRP3, inflammasome, Nrf2, inflammation, dentistry, oral immunological diseases, oral inflammatory diseases, oral microbiome

## Abstract

The aim of this review article was to summarize the functional implications of the nuclear factor E2-related factor or nuclear factor (erythroid-derived 2)-like 2 (Nrf2), with special attention to the NACHT (nucleotide-binding oligomerization), LRR (leucine-rich repeat), and PYD (pyrin domain) domains-containing protein 3 (NLRP3) inflammasome in the field of dentistry. NLRP3 plays a crucial role in the progression of inflammatory and adaptive immune responses throughout the body. It is already known that this inflammasome is a key regulator of several systemic diseases. The initiation and activation of NLRP3 starts with the oral microbiome and its association with the pathogenesis and progression of several oral diseases, including periodontitis, periapical periodontitis, and oral squamous cell carcinoma (OSCC). The possible role of the inflammasome in oral disease conditions may involve the aberrant regulation of various response mechanisms, not only in the mouth but in the whole body. Understanding the cellular and molecular biology of the NLRP3 inflammasome and its relationship to Nrf2 is necessary for the rationale when suggesting it as a potential therapeutic target for treatment and prevention of oral inflammatory and immunological disorders. In this review, we highlighted the current knowledge about NLRP3, its likely role in the pathogenesis of various inflammatory oral processes, and its crosstalk with Nrf2, which might offer future possibilities for disease prevention and targeted therapy in the field of dentistry and oral health.

## 1. Introduction

The innate immunity response constitutes the first line of a defense system against microorganisms, foreign substances, and endogenous defective cells. This system gets activated by unique microbial components, so-called pathogen-associated molecular patterns (PAMPS), or damage-associated molecular patterns (DAMPS, e.g., extracellular adenosine triphosphate—ATP, released from injured and dying cells), which are generated by endogenous stress. In response to these exogenous or endogenous stimuli, germline-encoded pattern-recognition receptors (PRRs) are triggered, which is the first line of host defense against microbial invasion [1]. To date, five different families of PRRs are known: extracellular Toll-like receptors (TLRs), AIM2-like receptors (ALRs), C-type lectin receptors (CLRs), DNA sensors, RIG-I-like receptors (RLRs), and NOD-like receptors (NLRs present in the cytoplasm with a central nucleotide-binding oligomerization domain (NOD) and a leucine-rich repeat (LRR) region) [1,2].

**Figure 1:**
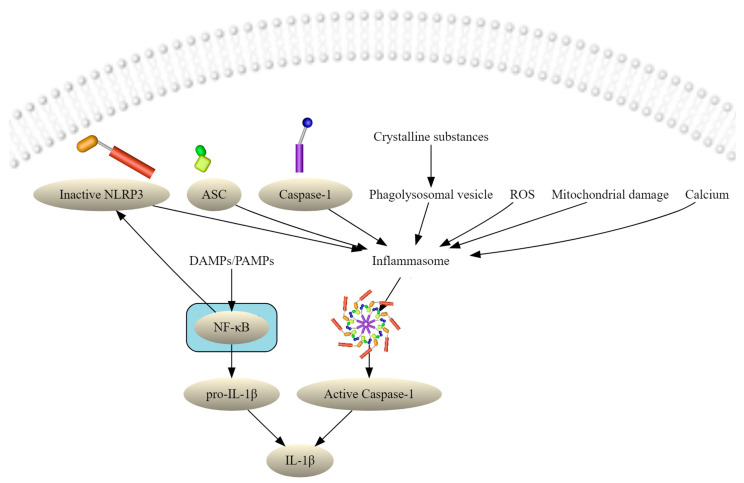
Schematic illustration of the NLRP3 inflammasome activation. The priming signal is provided by PAMPS, microbial ligands, or endogenous cytokines, which stimulate PRRs, leading to the transcription of NLRP3 and pro-IL-1β, due to the activation of the NF-κB pathway. The activation signal is provided by various DAMPs (e.g., ROS). Caspase-1 cleaves the pro-inflammatory cytokine pro-IL-1β. ASC: apoptosis-associated speck-like protein containing a CARD (caspase activation and recruitment domain). DAMPs: danger-associated molecular patterns. IL: interleukin. NF-κB: nuclear factor kappa B. PAMPs: pathogen-associated molecular patterns. ROS: reactive oxygen species.

PRRs are sensor protein components, triggering the activation of the nuclear factor-kappa-B (NF-κB) pathway, type I interferon (IFN), and other signaling pathways by defining so-called inflammasomes, which, in conclusion, play a crucial role in activating innate immunity and inflammation [2]. Furthermore, formation of the inflammasomes can also induce pyroptosis, an inflammation-related form of cell death [3,4,5,6,7]. The inflammasome, first described by Martinon et al. [8] in 2002, is a cytoplasmatic high-molecular weight protein complex serving as a platform of caspase-1 (CASP1) activation. The NACHT domain-, leucin-rich repeat-, and pyrin domain (PYD)-containing protein 3 (NLRP3) inflammasome complex (also known as NALP3 or cryopyrin) is related to the NLRs, and plays a crucial role as an innate immune sensor against microbial pathogens, including bacterial, fungal, or viral infections [9], and can sense a variety of stimuli, such as bacterial infection, extracellular ATP, crystals, and endoplasmic reticulum stress [10,11]. Immune cells like monocytes, macrophages, neutrophils, and dendritic cells express the NLRP3 inflammasome [12,13,14]. NLRP3 is a tripartite protein and contains an amino-terminal pyrin domain (PYD), a NOD, and a C-terminal LRR domain [15]. Beside the sensor molecule and the NLRP3 protein, the NLRP3 inflammasome complex also includes an apoptosis-associated speck-like protein containing CARD (ASC) and an effector protease, pro-caspase-1 (proCASP1) [16,17]. In conditions of rest, the NLRP3 complex is autoinhibited by an internal interaction between the NACHT domain and the LRRs, suppressing the interaction between NLRP3 and ASC [16]. For activation, the PYD domain mediates recruitment of ASC and proCASP1 to generate an active NLRP3 inflammasome complex [18] that stimulates activation and CASP1-catalyzed maturation and secretion of different inflammation-associated cytokines (IL-1β, IL-18, IL-33) in the extracellular milieu [19,20,21]. Pro-IL-1β is a potent proinflammatory cytokine and is processed by active CASP1 to mature IL-1β, as shown in Figure 1 [8,19,22].

Activation of the NLRP3 inflammasome complex requires two signals [10,11]:

(1) The priming signal is provided by PAMPS, microbial ligands, or endogenous cytokines, which stimulate PRRs, leading to the transcription of NLRP3 and pro-IL-1β, due to the activation of the NF-κB pathway. The second part of priming is the post-translational modifications (PTMs) that keep NLRP3 in an autosuppressed inactive but signal-competent state.

(2) The activation signal is provided by various DAMPs (e.g., reactive oxygen species—ROS). They promote NLRP3 oligomerization and recruitment of ASC and proCASP1, leading to the activation of the NLRP3 inflammasome complex (Figure 1).

For activation of the NLRP3 inflammasome, both signals are mandatory. Activated CASP1 then develops pro-IL-1β and pro-IL-18 to their mature and secreted forms, IL-1β and IL-18 [23].

Though several activation models are described, the exact molecular mechanism is still unknown. The pore formation and ion redistribution model depict the influence of ions, primarily the potassium imbalance as an NLRP3 activator [24]. Additional to disruption of lysosomes or mitochondria/metabolic balance, the noncanonical and one-step NLRP3 inflammasome activation via TLR4 stimulation is a well described pathway [25,26].

Next to downstream cytokine production, so-called pyroptosis, a rapid form of cell death associated with inflammation [27], can occur as a result of NLRP3 inflammasome activation. Key steps are the cleavage and recruitment of gasdermin D (GSDMD) and GSDMS^Nterm^, respectively. Once bound to phosphatidylinositol phosphates and phophatidylserine, proteins oligomerize and insert into the plasma membrane. After pore formation, cells enter pyroptosis.

NLRP3 has been the object of numerous studies in which gain-of-function mutations have been associated with various diseases characterized by the pathogenesis of inflammatory disorders, including gout, type 2 diabetes, and atherosclerosis [28,29]. The NLRP3 inflammasome was found to be involved in the development of gingival inflammation and alveolar bone loss [30], suggesting that NLRP3 plays a role in oral diseases.

The nuclear factor E2-related factor or nuclear factor (erythroid-derived 2)-like 2 (Nrf2), a member of the CNC-bZIP protein family, is a transcription factor and regulates cellular defense mechanisms. Under physiological conditions, Nrf2 interacts with Kelch-like ECH-associated protein 1 (Keap1) and is kept inactive in the cytoplasm [31]. In response to oxidative or inflammatory stress, Nrf2 is dissociated from Keap1 and transported to the nucleus where it induces the expression of cytoprotective genes, such as hemeoxygenase-1 (*HO-1*) and NAD(P)H quinone oxidoreductase-1 (*NQO1*), to defend against oxidative stress and inflammation [32,33], and to limit their detrimental effects [34]. Nrf2 induces the expression of cytoprotective genes as it regulates the transcription of antioxidant systems, ensuring the protection of Nrf2-activated cells during inflammation [35,36] via its anti-inflammatory properties [37].

**Figure 2:**
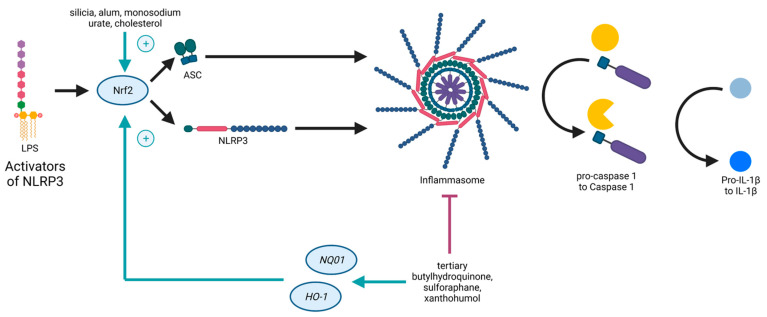
Schematic illustration of the crosstalk between Nrf2 and the NLRP3 inflammasome. NLRP3 (nucleotide-binding oligomerization domain (NOD)-like receptor containing pyrin domain 3) inflammasome activation causes Nrf2 degradation. NLRP3 inflammasome inhibition by Nrf2 activation upon Nrf2-activating compounds. Nrf2 activated by, e.g., cholesterol crystals, promotes NLRP3 inflammasome activation.

Both the Nrf2- and the NLRP3-pathways are inflammation-associated, stress-induced, and linked to ROS, as well as to NF-κB. On the one hand, ROS is supposed to induce NRLP3 inflammasome activation [38]. On the other hand, genes expressed due to Nrf2 detoxify ROS [39]. Nrf2 contributes to NLRP3 inflammasome activation under the condition of oxidative stress [40]. As mentioned before, NF-κB is necessary for the priming signal of NLRP3 inflammasome activation and also leads to Nrf2 expression [36]. Furthermore, it was shown that the pathways of Nrf2 and NLRP3 are interconnected in an antagonistic manner [31], as Nrf2 activation by Nrf2-activating compounds (such as tertiary butylhydroquinone, sulforaphane, and xanthohumol) is accompanied with NLRP3 inflammasome inhibition [41], providing evidence for novel treatment options against inflammatory disorders. Studies demonstrated that NLRP3 inhibition due to Nrf2 activation is accompanied with a reduction of NF-κB activation [42,43]. Carbon monoxide, generated in the catalysis of *HO-1*, is a negative regulator of NLRP3 inflammasome activation [44], and thus, inhibits pyroptosis [45]. However, Nrf2 activated by cholesterol crystals or monosodium urate crystals promotes the activation of the NLRP3 inflammasome [41] (Figure 2).

Overall, activation of the host immune response, and further, of inflammation play a crucial role in the development of many chronic diseases. As a pathophysiologic starting point of these processes, several intracellular multimeric protein complexes that activate inflammatory cascade-inducing caspases, the inflammasomes, were identified. There has been recent progress in understanding the role of the NLRP3 inflammasome in oral and systemic diseases. In the field of dentistry, however, evidence regarding the effects of this inflammasome and its potential inhibition, as well as activation due to Nrf2, is missing.

In this review, we critically examine the role and potential therapy approach of the NLRP3 inflammasome complex linked to dental medicine, regarding its crosstalk to Nrf2.

## 2. The Oral Microbiome

As the initial part of the digestive tract, the oral cavity is the first part of the human microbiome and is followed by the esophagus, stomach, intestine, and colon. More than 700 bacterial, archaeal, viral, and fungal species inhabit the oral cavity and provide the so-called oral microbiome [46,47,48].

With the achievement of the Human Microbiome Project in 2008, the role of the microbiome in human diseases has become attractive for scientists. Most of the microbiome research focused on the gut microbiome at first, but studies on other organs, i.e., the oral cavity, are increasing progressively. Especially in the field of dentistry, the oral microbiome is a fundamental part of good dental and oral health. With the Human Oral Microbiome Database (HOMD; www.homd.org; 01/09/2022), extensive information on the approximately 700 predominantly bacterial species of the oral cavity should be provided to the scientific community.

Among digestive organs, the oral cavity is a place of tremendous heterogeneity, due to the presence of teeth and various tissue compositions. Thus, many different niches, including surfaces of the teeth, tongue, cheeks, palate, and tonsils, occupied by specific organisms, form varied environmental compositions with different functional characteristics [46,49,50]. Dental biomaterials, or prostheses and implants create supplementary locations for biofilm formation [51,52,53]. In addition, the divergence of the composition of each niche is positively correlated with the periodontal pocket depth and periodontitis progression [54].

As one of the first bacterial compositions, dental plaque has been described as a complex, polymicrobial, and highly structured biofilm [55,56]. In the 20th century, researchers discovered species of *Streptococcus mutans* [57], which are usually the first pioneering microorganisms of the oral cavity, and the specific periodontal pathogens *Aggregatibacter actinomycetemcomitans* (*A. actinomycetemcomitans*) [58], *Porphyromonas gingivalis* (*P. gingivalis*), *Treponema denticola*, and *Tannerella forsythia* (*T. forsythia*) [59].

Before birth, the toothless oral cavity of a fetus is sterile and becomes colonized by a common bacterial flora by passing through the birth canal [60,61]. With the eruption of teeth, a new habitat for microorganisms occurs due to the teeth themselves, and furthermore, through the gingival crevice, which is nourished by the gingival crevicular fluid.

Key to a healthy oral cavity, according recent studies, are microorganisms that can be classified into six phyla, i.e., *Firmicutes, Bacteroidetes, Proteobacteria, Actinobacteria, Spirochaetes*, and *Fusobacteria* [62,63,64].

In healthy individuals, a dynamic balance between the microbiome and the host produces important benefits, i.e., controlling the cardiovascular system, defending against potential pathogens, maintaining a healthy digestive tract, developing and supporting host defense functions, and having anti-inflammatory properties [49,65]. If this homeostasis fails, prolonged dysbiosis and chronic inflammation develop, potentially leading to microbial changes and inflammatory disease complications. To provide oral health of the microenvironment in the biofilm on teeth, the degree of inflammatory response of the tissues in contact with these biofilm is of great importance [66].

According to recent studies, an imbalance or dysbiosis of the oral microbiome is related to dental caries or periodontitis [67], oral cancer [68], inflammatory bowel disease [69], and systemic diseases, including Alzheimer’s disease [70], cardiovascular diseases [71], atherosclerosis [72], and colorectal, pancreatic, and other cancers [73,74,75].

A potential microbiome procarcinogenic mechanism derives from TLR-mediated microbial pattern recognition. As TLRs play a crucial role in the interplay between the microbiome and immunity, they stimulate proinflammatory signaling pathways via NF-κB activation [76], induced by oral microbiota dysbiosis [72]. As NF-κB leads also to Nrf2 activation [36], it might be hypothesized that Nrf2 is critical for inhibiting an exaggerated immune response leading to inflammatory diseases.

Furthermore, it has been speculated that Nrf2 has protective effects against inflammatory bowel disease, a chronic and inflammatory disease of the gastrointestinal tract [77].

**Figure 3:**
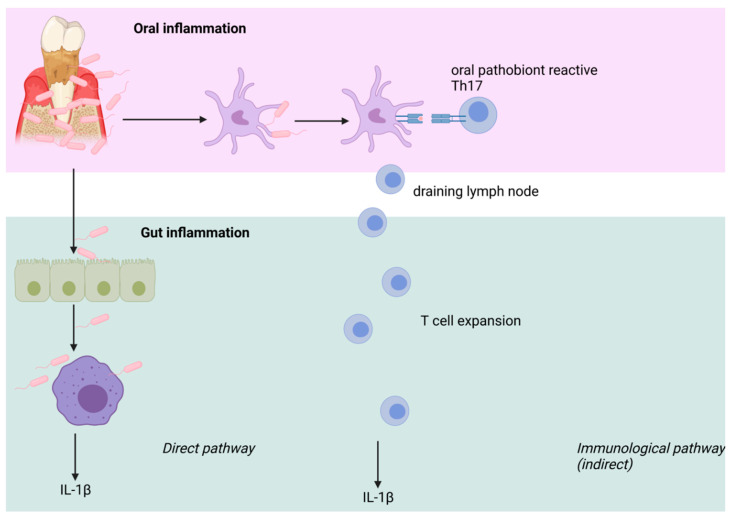
Schematic illustration of immunological pathways of the oral microbiome. There is a direct and an indirect immunological pathway for dysregulations of the oral microbiome effecting the colonic microbiome.

Poor oral hygiene facilitates bacterial translocation to other body sites by transmigration from oral draining lymph nodes to other lymphoid organs, i.e., the gut [78], which means that the oral microbiome and colonic microbiome are physically connected [79]. (Figure 3). Nrf2 is known to be part of gut development [80] and maintains its regular functionality [33].

Inflammation, by the specific activation of the NLRP3 inflammasome, is an important contributor to the development of atherosclerotic cardiovascular disease. Baragetti et al. [81] evaluated this complex pathway to be activated by a number of factors, such as unhealthy nutrition, altered gut, and changed oral microbiome composition. 

Moreover, a relevant role of oral microbiome dysbiosis has recently been demonstrated, since it can induce systemic diseases and worsen the metabolic parameters of chronic diseases. Additional studies are expected to confirm that an oral anti-inflammatory treatment and improved oral hygiene can reduce the incidence of diseases. In addition, it is of interest whether the anti-inflammatory effect of Nrf2 may affect the oral microbiome in a protective manner or not.

Overall, innovative development of molecular genetics in DNA sequencing approaches enables us to gather fundamental issues of the oral microbiome and its association with oral and systemic health. Further studies may lead to promising dental interventions to manage the bacterial composition of the oral microbiome, as it has been viewed as an important factor affecting human microbiota.

## 3. Periodontitis

Periodontitis (PD) is a common chronic inflammatory disease that is caused by bacterial infection in the subgingival microbiome and affects the periodontal tooth-supporting tissues of select teeth or rarely the entire oral structure (gingiva, periodontal ligaments, and alveolar bone). Due to the persistence of periodontal pathogens and an imbalance of the immune response that they encode, PD is characterized by periodontal attachment loss, bone resorption, and can finally lead to tooth loss [82]. Besides tooth loss, PD can influence systemic health, when oral microorganisms enter the bloodstream by crossing damaged oral mucosa [72]. Consequently, PD could affect systemic diseases, i.e., cardiovascular disease [83], rheumatoid arthritis [84], type 2 diabetes [85], and cancer [86]. The main function of the human immune system is to differentiate between commensal bacteria, related to a commensurately oral microbiome, and pathogenic bacteria. Thus, in a healthy balance and established homeostasis, common microorganisms interact with the immune system without provoking a proinflammatory response [87].

The most likely cause for PD to occur is due to an accumulation of pathogenic bacteria on the tooth surfaces and in the gingiva, followed by inflammation [30] caused by activation of signaling pathways in PRRs [20], and thereby, generation of proinflammatory cytokines. In recent years, studies have shed light on the processing steps of these cytokines. Inter alia, this process is dependent upon an intracellular innate immune sensor, the NLRP3 inflammasome that also might influence the PD activity, in response to various bacterial, physical, and chemical agents [30,88,89].

The IL-1 family of cytokines, such as interleukin-1β (IL-1β) and interleukin-18 (IL-18), are proinflammatory cytokines, which are involved in the pathogenesis of several bone-affecting inflammatory diseases. Furthermore, they mediate bone loss when produced unbalanced. An unbalanced production is due to higher recruitment and differentiation of osteoclasts in the tissues via activation of the receptor activator of nuclear factor kappa-B ligand (RANKL) in osteoblasts [21,90]. Above all, as an osteoclastogenic factor, RANKL regulates the formation and activity of osteoclasts and upregulates alveolar bone loss [91]. It has been demonstrated that NLRP3 deficiency can significantly decrease RANKL, indicating the relevance of the NLRP3 inflammasome in supporting osteoclast-genesis in PD [20]. Studies demonstrated IL-1β to be a potential marker of this disease, due to its higher level in serum, saliva, and gingival tissue of PD patients [92,93,94]. In the gingival tissue and crevicular fluid of patients with PD, increased expression of IL-1β and IL-18 has been positively correlated with increased expression of *NLRP3* mRNA [30]. Moreover, an increased expression of *NLRP3* mRNA in the oral epithelium [95] and in the saliva [96] of patients has been found with the simultaneous downregulation of NLRP3 inhibitors [95,97]. Many studies demonstrated that the NLRP3 inflammasome is involved in the development of gingival inflammation and subsequent bone loss, due to an exaggerated immune response [20,30,88,95,96,98]. In a murine model with bacterial plaque-retentive ligatures placed around the teeth, Marchesan et al. [99] further support the NLRP3 upregulation in experimental periodontitis.

In response to several bacterial ligands acting as PAMPs, e.g., lipopolysaccharide (LPS) [14,100], peptidoglycan [19], bacterial and viral RNA [9,101], and flagellin [4], NLRP3 regulates the maturation and secretion of proinflammatory cytokines, like IL-1β [102] via proCASP1. Furthermore, an upregulation of the NLRP3 inflammasome complex leads to an increase in the genesis of IL-1β [30,97] and IL-18 [88,103,104].

IL-1β is mainly produced in monocytes, which are declared to express *NLRP3* mRNA [105,106]. Sutterwala et al. [14] found that this process was highly induced by bacterial LPS. On the other hand, there was no production of IL-1β in NLRP3-deficient macrophages, even though bacterial stimulations occurred [19,23,101,107].

Treatment with an IL-1 receptor antagonist of patients with rheumatoid arthritis cancelled clinical symptoms, suggesting a cause–effect relationship between IL-1β production and the development of the disease [108]. Based on an inhibition of the NLRP3 inflammasome, some studies also presented therapeutic pathways in the treatment of experimental PD [109,110].

Moreover, Nrf2 has been demonstrated to directly suppress transcription of NLRP3-associated genes, including *pro-IL-1β*, and *pro-IL-1α* [111,112], suggesting Nrf2 to be a potential therapeutic inhibitor of PD.

Delineating the likely role of several oral microbiota associated with the development of PD is rather complex. Among the thousands of bacterial species in the oral cavity, few Gram-negative anaerobic bacteria were related to PD genesis. Periodontopathogen species dispose of several virulence factors that allow them to survive in the host environment by selectively adapting the host’s immune-inflammatory response. The “red cluster” of periodontopathogenic bacteria, consisting of *P. gingivalis*, *Treponema denticola*, *Prevotella intermedia*, *A. actinomycetemcomitans*, and *T. forsythia*, contribute to the initiation and progression of severe PD [113,114].

Teles et al. [115] showed a positive correlation between these bacterial species and the overexpression of proinflammatory cytokines, i.e., IL-1β and IL-18. Consequently, as PAMPS, these bacteria are attributed to have an impact on the etiology and progression of PD by activating inflammasome activity and controlling the NLRP3-mediated inflammatory response in PD [116]. Furthermore, an in vitro study has demonstrated periodontopathogenic bacteria, such *P. gingivalis*, *A. actinomycetemcomitans*, or *Fusobacterium nucleatum (F. nucleatum)*, to be responsible for an increased expression of NLRP3 [117]. Several signaling pathways have been demonstrated to generate and promote the occurrence of PD. In this context, it is essential to know and understand them, as modulating them may be the key in preventing or treating PD.

Therefore, we want to present and discuss representative periodontal pathogens, which play a crucial role in activating inflammation in PD, with special attention to the roles of NLRP3 and Nrf2.

### 3.1. P. gingivalis

*P. gingivalis*, a Gram-negative, nonmotile, anaerobic oral bacterial species, is a prominent component of the subgingival microbiome [118], and is the key etiological agent in PD [119]. *P. gingivalis* leads to a state of bacterial dysbiosis and, as a major periodontal pathogen, it is the origin of chronic PD genesis [120].

Several virulence factors are responsible for *P. gingivalis* survival and evasion from the host’s immune system, i.e., LPS, outer membrane vesicles (OMVs), fimbria, nucleoside diphosphate kinase, and ceramide [121]. LPS is a component of *P. gingivalis* and appears in two versions: penta-acylated LPS and tetra-acylated LPS [122]. LPS, as a virulence factor and so-called priming signal, is responsible for the generation of NLRP3, and subsequently, pro-IL-1β and pro-IL-18 by promoting Toll-like, receptor-dependent signaling [123,124], which triggers the NF-κB pathway [125]. When phosphorylated, due to the impulse by LPS, NF-κB connects to the binding sites in the NLRP3 promoter region, resulting in the NLRP3 inflammasome activation in immune cells [126], which was greatly associated with periodontal damage [127] and bone loss due to increased IL-1β production [128,129]. Furthermore, studies determined the presence of IL-1β and IL-6 in periodontal tissues, after gingival epithelial cells were exposed to LPS [130,131].

In a murine model of *P. gingivalis* infection on NLRP3 and absent in melanoma 2 (AIM2)-depleted mice, Okano and colleagues [128] demonstrated that secreted or released factors from *P. gingivalis* activate NLRP3, rather than the AIM2 inflammasome, in bone marrow-derived macrophages. Besides the in vivo assay of this study, the authors also performed an in vitro study on human monocytic cells (THP-1). In both human cells and mouse macrophages, LPS-induced priming is required for IL-1β release, but this dependency is higher in mouse macrophages than in THP-1 cells. This confirmed the first study’s outcomes from Chiang et al. [132], where IL-1β deficient mice showed less *P. gingivalis* LPS-induced destruction of the periodontium by contrast with wild-type mice treated equally.

Besides LPS, it has been shown that OMVs shed from *P. gingivalis* trigger inflammasome activation, as well. Macrophages were stimulated in vitro and in vivo by ASC speck formation, as displayed by IL-1β release [133,134]. Moreover, in human THP-1 cells, the OMVs of several periodontopathogenic bacteria, i.e., *P. gingivalis*, *T. denticola*, and *T. forsythia,* can provoke the production of proinflammatory cytokines, due to the NF-κB pathway, and consequently, lead to the activation of NLRP3 and AIM2 inflammasomes.

Yang et al. [135] further demonstrated a synergy between LPS from *P. gingivalis* and hypoxia. They determined that a single stimulation of human gingival fibroblasts by one of those two priming signals did not affect NLRP3 inflammasome activation, but the combination of both did. Subsequently increasing IL-1β levels and pyroptosis are relevant in causing gingival inflammation, which, in conclusion, is essential for the pathogenesis of PD. The current literature has extensively reported on the role of NLRP3 in PD. In consequence, these data indicate that NLRP3 plays a significant role during PD pathogenesis and further studies should shed light regarding this aspect.

It can be pointed out that inflammasome activation in macrophages infected with *P. gingivalis* may also promote inflammatory bone destruction via release of proinflammatory cytokines, i.e., IL-1β. A highly positive correlation between the severity of PD and increased levels of IL-1β [136] suggests a stimulation of osteoclast formation, directly or indirectly, via NLRP3 inflammasome activation.

In conclusion, the role of *P. gingivalis* in the occurrence of PD is of great importance, regarding potential strategies for PD treatment. Thus, regulating the inflammatory response by interfering with the inflammasome activation pathway may become a potential strategy for PD treatment.

Li et al. [109] showed that 1,25-dihydroxyvitamin D3 (VD3) regulates the inflammatory response in PD via modulating the Aryl hydrocarbon receptor (AhR)/NF-κB/NLRP3 inflammasome signaling pathway in a murine model. Furthermore, they determined that the expression of NLRP3, ASC, and CASP-1 was increased in the gingival epithelium in PD, while it was decreased upon VD3 treatment.

Another potential strategy for PD treatment might be the activation of the Nrf2/*HO-1* pathway, which reduces the inflammatory response of human gingival fibroblasts (HGFs) [137] and macrophages [138] stimulated by LPS from *P. gingivalis*. Additionally, Huang et al. [139] found that ED-71, a vitamin D analog, reduced LPS-induced ROS levels, the activation of the NLRP3 inflammasome, and eventual pyroptosis by activating the Nrf2/*HO-1* pathway.

### 3.2. Fusobacterium Nucleatum

*F. nucleatum*, a Gram-negative and anaerobic bacterial species, is one of the first to become established in the dental plaque biofilm among abundant microorganisms during PD [140]. As one of the first periodontopathogenic bacteria in line, it stimulates inflammation in the tissues of the oral cavity, and represents one of the most important pathogens that lead to PD [140], due to inducing apoptosis of immune cells and bone loss [141,142]. With the inflammatory progression and severity of PD, the prevalence of *F. nucleatum* increases [143]. Like other periodontopathogenic bacteria, *F. nucleatum* also presents virulence factors, i.e., adhesins [143], endotoxins [144], and serine proteases [145]. Its ability to connect with several other oral microorganisms by producing adhesins makes it a bridge that connects former colonizers and late pathogens, which results in the development of dental plaque [146]. In consequence, lower levels of *F. nucleatum* are associated with lower levels of late colonizers [147]. *F. nucleatum* is reported to promote the growth of *P. gingivalis* [148], and may also support the invasion of *P. gingivalis* and *A. actinomycetemcomitans* in human gingival epithelial cells [149]. When invaded into human tissues, *F. nucleatum* may interfere with or promote recovery processes of already damaged periodontal tissues [150,151].

Studies described NLRP3 inflammasome activation and IL-1β secretion due to *F. nucleatum* infection in murine macrophages [152], and in gingival epithelial cells due to the activation of the NF-κB signaling pathway [104], even in the absence of extracellular ATP. Consequently, it may be indicated that, unlike *P. gingivalis*, *F. nucleatum* delivers PAMPs and DAMPs.

Hung et al. [153] proposed that, in gingival epithelial cells during *F. nucleatum* infection, NLRX1 (NLR family member X1) is able to enhance the host immune response due to periodontopathogen infection via the NLRP3 inflammasome, but simultaneously works as a guardian preventing uncontrolled inflammation during standard homeostasis status.

In addition, *F. nucleatum* plays an important role in the development of colorectal cancer, and was shown to promote metastasis by the TLR4/Keap1/Nrf2 axis [154].

### 3.3. Aggregatibacter actinomycetemcomitans

*A. actinomycetemcomitans* is also a Gram-negative bacterial species, first identified as a possible periodontal pathogen in 1976 [155], associated with the rapid progression of PD in adolescents [156,157], and localized in aggressive PD [158]. It colonizes the oral biofilm in later stages and invades the periodontal pocket’s epithelium [159].

As part of the HACEK group of Gram-negative organisms, *A. actinomycetemcomitans* is identified as causing infective endocarditis [160]. Furthermore, it may be associated with other systematic diseases, i.e., pericarditis [161], pneumonia when aspirated [162], as well as cardiovascular disease and arthritis [163,164].

The dysbiosis induced by *A. actinomycetemcomitans* is owed to its virulence factors, such as leukotoxin (Ltx) and cytolethal distending toxin (Cdt) [103]. Ltx was shown to kill human leukocytes via apoptosis or lysis [165]. Studies have examined that *A. actinomycetemcomitans* also mediates NLRP3 inflammasome activation in human mononuclear leukocytes [103,166], human osteoblastic cells [167], THP-1 monocytes [166], and murine macrophage-like cell lines [168]. Moreover, *A. actinomycetemcomitans* promotes apoptosis of human osteoblasts at least partially via NLRP3 inflammasome activation [167]. While *A. actinomycetemcomitans* enhanced the expression of NLRP3, TLR4, TLR2, and NOD2 in macrophages, which secrete IL-1β [169,170] and IL-18, virulence factors did not have an effect on the production of proinflammatory cytokines in human gingival epithelial cells (HGEC) [171,172,173]. As the first line of the human defense barrier, HGECs are a barrier against periodontal pathogens in oral tissues; thus, the missing response to the virulence factors of *A. actinomycetemcomitans* may determine a possibility for evading host defense.

To our knowledge there are no studies regarding the potential relationship between *A. actinomycetemcomitans* and Nrf2.

## 4. Periapical Periodontitis

Besides PD in the traditional sense of term, i.e., gingival PD, periapical PD is one of the most common inflammatory diseases in adults. In response to caries, tooth fracture, or trauma, oral microorganisms can enter the initial sterile tooth pulp and trigger inflammation, which may result in pulp necrosis [174,175]. Symptoms are varied, implicating sensitivity to pressure or cold, pain, periapical radiolucency, and edema [176]. There is a distinction between acute and chronic periapical PD showing different symptoms [175]. Most of endodontic bacteria are located in the root canal [177]; thus, the therapy of choice is a root canal treatment, aiming to remove the inflamed dental pulp [178,179]. Surgical apicoectomy is required when endodontics is insufficient and the inflamed part of the bone includes the tooth apex [180]. Etiology of this odontogenic infection is due to bacterial species and their virulence, as well as the interaction with immunological host responses [175]. It was shown that apical PD is responsible for generating cytokines by recruiting inflammatory cells, i.e., host immune response to inflammatory processes [181].

The most common pathogen in periapical PD was demonstrated to be *Enterococcus faecalis* (*E. faecalis*), a Gram-positive coccus [182,183,184]. It was already shown that *E. faecalis* is able to promote CASP1 activation and pro-IL-1β expression, which subsequently increases IL-1β levels [185]. Moreover, increasing IL-1β production during periapical PD [186] might be associated with an interplay between this inflammatory disease and the NLRP3 inflammasome.

Studies demonstrated that one virulence factor of *E. faecalis*, i.e., lipoteichoic acid (LTA), activates the NLRP3 inflammasome through the NF-κB signaling pathway, and further, leads to IL-1β secretion via upregulation of ROS [187]. Therefore, it has been speculated that the inhibition of ROS may regulate periapical PD. In a pursuing study, Yin et al. [182] examined Dioscin, an antioxidative drug [188] with antibacterial and anti-inflammatory effects [189], as an inhibitor of LTA-mediated NLRP3 activation in mouse macrophages. Results also indicated a positive correlation between inflammasome activation and decreased osteoblast activity in periapical PD. Hence, further studies are necessary to confirm Dioscin as a potential root canal sealant for the treatment of periapical PD.

Former studies already approved the presence of the NLRP3 inflammasome signaling pathway in periapical PD and connected its deterioration and inflammatory intensity with increased NLRP3 levels [190,191]. Moreover, inflammasomes are known to induce pyroptosis, which is responsible for the destructive effects of periapical PD. The occurrence of pyroptosis in periapical PD was indicated when pyroptosis was significantly increased in rats with acute periapical periodontitis and subsequent bone loss [192]. However, during CASP1 inhibition, pyroptosis was moderated, indicating a positive correlation between pyroptosis levels to the degree of inflammation in periapical PD. Ran and colleagues [193] further confirmed that *E. faecalis* and its virulence factors increase GSDMD processing in THP-1 macrophages, resulting in pyroptosis due to the activation of the NLRP3 inflammasome. Furthermore, Guan et al. [194] revealed a positive correlation between NLRP3 activity and estrogen-mediated periapical PD in postmenopausal patients and ovariectomized rats, suggesting that NLRP3 is responsible for the consequent bone resorption during this disease.

Additionally, a fungal species is also related to periapical PD: *Candida albicans*. It was shown that it also leads to pyroptosis by activating the NLRP3 inflammasome in mononuclear phagocytes and macrophages [195]. In addition, LPS from *P. gingivalis* is known for inducing CASP1-mediated pyroptosis in human dental pulp cells [192]. As human dental pulp cells are the main component of the dental pulp fluid [196], several studies have investigated the pulpal innate immune response by the NLRP3 inflammasome pathway. Song et al. [197] were the first to determine the mRNA of NLRP3 in human dental pulp cells, although the role and function of the NLRP3 inflammasome in human dental pulp cells remained unclear. Later, it was confirmed that the NLRP3 inflammasome is involved in the occurrence of dental pulp inflammation. The stronger the inflammation, the higher were the mRNA expression levels of NLRP3 and subsequent IL-1β secretion. Moreover, silencing the NLRP3 gene induced a decrease in cytokines. These results indicate that local inhibition of NLRP3 may reduce the impact of cytokine-mediated, host-destructive processes in pulpitis [198]. Furthermore, it was ascertained that the expression of NLRP3 in human dental fibroblasts varies in different degrees of periapical PD, showing higher NLRP3 levels in irreversible pulpitis, in contrast to reversible pulpitis [199].

The TLR4/NF-κB pathway was demonstrated to be related to the activation of the NLRP3 inflammasome in LPS-stimulated human dental pulp cells [200].

Wang et al. [201] investigated the effect of miR-223 on NLRP3 in human dental pulp fibroblasts, assuming that miR-223 plays a key role in the regulation of host immune responses [202]. They determined an upregulation of NLRP3 when reversible pulpitis evolved into irreversible pulpitis, and miR-223 was considered to be an inhibitor of this signaling pathway.

Taken together, penetration of several bacteria into the dental pulp leads to inflammatory responses in dental pulp cells, attributing a main function to the NLRP3 inflammasome.

## 5. Oral Squamous Cell Carcinoma

With an incidence of 90%, oral squamous cell carcinoma (OSCC) is the most common oral cancer [203] with a low 5-year survival rate of only 30% [204]. Despite the frequent inspection of the oral cavity by dentists assuming patients’ responsibility for oral health, most OSCC lesions were missed at an early stage, which may explain the high mortality rate. OSCC is known to occur three times more frequently in men than in women [205]. Besides smoking and alcohol consumption [206], risk factors of OSCC also include chronic inflammation [207]. Several studies have shed light on inflammation as a possible cancer basis, as Hussain et al. [208] already determined in 2003 that inflammation is the cause of one in four cancers. Furthermore, oral bacterial species are reported to be responsible for these inflammatory disorders via influencing key processes, which may be contributing to oral carcinogenesis [209,210].

As already described before, *P. gingivalis* and *F. nucleatum* induce the development of proinflammatory cytokines, at least partially, through the NLRP3 pathway. Therefore, it was proved that these periodontopathogenic bacteria are potential etiological agents for oral cancer [211]. Yang et al. [212] provided evidence that *Fusobacteria* can be associated with cancer staging of OSCC. Interestingly, Tezal et al. [213] evaluated patients whom had never used tobacco and alcohol, but suffered from PD. These patients showed a higher probability of 32.8% for poorly differentiated OSCC than patients of good oral health at 11.5%. A very recent study by Yao et al. [214] from 2021 developed a new mechanism connecting periodontopathogenic bacteria (*P. gingivalis* and *F. nucleatum*) and OSCC, by showing that these bacterial species upregulate NLRP3 expression and drive inflammasome activation. It was examined that this pathway can level up tumor growth and tumor proliferation. *P. gingivalis* has been reported to be carcinogenic. Studies demonstrated a positive correlation between *P. gingivalis* infection and size or invasiveness [143,144], as well as a late tumor–node metastasis (TNM) stage, and low differentiation [145] of head and neck squamous cell cancer (HNSCC). In addition, Sinha et al. [215] first demonstrated that *P. gingivalis* is enriched in feces from patients with colorectal cancer. Wang et al. [216] later confirmed that *P. gingivalis* can be held responsible for enhancing colorectal cancer, due to the activation of the NLRP3 inflammasome.

Taken together, oral health and, subsequently, the oral microbiome and its interplay with the host immune response may play a critical role in the development of OSCC. The importance of an efficient and healthy oral microbiome has been underlined by developing a novel strategy of detecting OSCC due to the utilization of saliva, which makes the oral microbiome a noninvasive diagnostic tool [149,212].

Standard therapy of OSCC is a treatment using 5-Fluorouracil (5-FU), which inhibits pyrimidine metabolism and DNA synthesis. It was determined that treatment with 5-FU leads to increased intracellular ROS and is ascertained to upregulate NLRP3 and IL-1β expression in human OSCC cell lines. This subsequently mediates a chemoresistance of OSCC to 5-FU lying on multiple aspects, suggesting tumor-associated macrophages to be responsible. Moreover, survival rates of patients decreased with higher NLRP3 expression, and NLRP3 deficiency enhanced the antitumor effect of 5-FU [217]. This might confirm that NLRP3 is responsible for not only the progression of OSCC but also its limited treatment effectiveness.

As NLRP3 is likely to be critically involved in OSCC occurrence, progression, and proliferation, few studies have shed light on possible strategies for oral cancer treatment, regarding the NLRP3-inflammasome. MicroRNAs are known to act as regulators of carcinogenesis in general. Feng et al. [218] implicated microRNA-22 to inhibit OSCC proliferation due to interference of the NLRP3 pathway.

So-called dietary exosome-like nanoparticles extracted from mushrooms [219] or ginger rhizomes [220] were identified as inhibitors of the NLRP3 inflammasome.

Yang et al. [221] showed that also bitter melon-derived extracellular vesicles (BMEVs) may downregulate the NLRP3 activation and, moreover, reduce the drug resistance of 5-FU. Additionally, BMEVs could inhibit OSCC proliferation due to the development of reactive oxygen species.

BAY 11-70082 is a sulfonic derivative and an inhibitor of NF-κB, known for having anticancer and anti-inflammatory effects [222]. Scuderi et al. [223] found that BAY 11-70082 could downregulate NLRP3 activation, and further, reduce tumor mass in mice.

In summary, OSCC treatment targeting NLRP3 is highly promising, and thus, necessitates further research.

It is known that oxidative stress plays an important role in the development of cancer. Nrf2, as an oxidative stress marker, was found to be associated with carcinogenesis and progression of OSCC [224] when hyperactive, while it inhibited carcinogenesis of normal cells [225]. This might suggest a prognostic value of Nrf2 and its potential role as therapeutic target.

## 6. Dental Implants

Today, the use of dental implants is indispensable in the treatment of edentulism. For the success and persistence of an implant, a connection between implant and living bone tissue is needed. Unlike a natural tooth, which is bound to the surrounding bone indirectly by the periodontal ligament, implants are directly engaged to the bone [226]. Implant stability can be divided into an early stage due to mechanical alliance to the bone, and secondly, into a stage of stability depending on regeneration and remodeling of the bone and tissue close to the inserted implant [227], named osseointegration [228]. Overall, the interaction between bone, tissues, implant surface, and the host immune response has to be compensated for, revealing true osseointegration [229]. Trindade et al. [230] confirmed that titanium implants activate the immune system and lead to inflammation, indicating a two-step osseointegration: first, recognition of the implant as a foreign body; second, development of a bone-forming environment to shield the foreign material from host tissues.

Once again, this shows the importance of a healthy and balanced interplay between the oral microbiome and the immune response, as criteria for implant success and in avoidance of uncontrolled inflammation leading to bone loss and subsequent loss of the implant. Despite advanced technology, failure of implantation (around 1.9–3.6% of dental-implant subjects) and subsequent loss of the implant cannot be ruled out [231]. Besides triggering factors such as medication [232], increasing prevalence of bad systemic health with higher age (≥75 years) [233], or smoking [234], the fundamental reason for implant failure is known to be an overreaction of the immune system, leading to bone loss [235]. Pathogen invasion from the implant surface structure [236], or bad oral hygiene [237] constitute a potential trigger for inflammation, and further, genesis of periimplantitis.

Periimplantitis is an irreversible disease characterized by inflammation of the supporting bone and connective tissues surrounding a dental implant, resulting in unsuccessful osseointegration and subsequent implant failure [238]. A systematic review from Rakic and colleagues [239] in 2018 showed a prevalence of periimplantitis in 12.8% of all implants used. Another study from 2019 revealed that 1/3 of all patients and 1/5 of all implants underwent periimplantitis [240]. Moreover, it has been shown that the incidence of periimplantitis increases with implant age [241].

Studies showed that proinflammatory cytokines are expressed at higher concentrations in the crevicular fluid of healthy implants than around teeth [242]. Furthermore, levels of proinflammatory cytokines in the peri-implant crevicular fluid are again higher around implants with periimplantitis than around healthy implants [243]. Many studies associated IL-1β to with playing a critical role in the occurrence of periimplantitis [244] and peri-implant bone loss [245], which is similar to PD, suggesting that the NLRP3 inflammasome plays, at least, a partial role.

Titanium implants release Ti ions into surrounding tissues [246], which further leads to the secretion of IL-1β, TNF-α, and RANKL in Jurkat T-cells [247], and might aggravate inflammation. Li et al. [248] confirmed these facts, and further, showed that Ti ions activate the NLRP3 inflammasome, increasing the release of ROS.

*Candida species* were found to be associated with periimplantitis [249] and triggered the NLRP3 inflammasome-mediated pyroptosis in macrophages [250]. As mentioned before, LPS from *P. gingivalis*—another bacterial species related to periimplantitis— also activates NLRP3 [191].

Taken together, inflammasomes, and mainly the NLRP3 inflammasome, may play a critical role in the development of periimplantitis. Considering the absence of therapeutic agents for the treatment of periimplantitis, further studies are needed to follow up on targeting inflammasome pathways as a future therapeutic option.

We suggest further molecular biologic studies be undertaken on interactions between dental implants and Nrf2.

## 7. The Alveolar Bone

It is already confirmed that the NLRP3 inflammasome attenuates osteogenesis by mediating inflammation and inducing osteoblast pyroptosis due to the processing of GSDMD and CASP1, and the following secretion of proinflammatory cytokines [251]. As NLRP3 affects bone homeostasis through the regulation of osteoclasts, osteoblasts, and other cell types, one might suggest that NLRP3 plays a vital role in the metabolism of the alveolar bone. However, due to unbalanced NLRP3 activation, alveolar bone homeostasis is disrupted, leading to local dysregulations, or acting as a foundation for systematic bone diseases [182,251]. Qu et al. [252] demonstrated hyperactive NLRP3 expression in osteoclasts, encouraging osteolysis in the absence of systemic inflammation. Furthermore, uncontrolled activation of NLRP3 is associated with osteopenia, a preliminary stage of osteoporosis [253,254].

Activation of the NLRP3 inflammasome in macrophages due to *P. gingivalis* infection or related to bisphosphonate therapy may also lead to bone loss due to increased IL-1β production [128,129]. Aging, estrogen deficiency, or hyperparathyroidism provide a chronic inflammatory microenvironment and enhance NLRP3 activation, which can further lead to bone resorption and genesis of osteoporosis [254,255,256,257].

On the one hand, Zang et al. [258] already stated that the inflammasome mediates age-related alveolar bone loss, on the basis of a correlation between alveolar bone loss in aged mice and elevated levels of IL-1β. On the other hand, NLRP3-deficient mice demonstrated improved bone mass qualities, presented by an increased bone density [253,258]. Treatment with an inhibitor of NLRP3, i.e., MCC950, significantly suppressed alveolar bone loss [258]. Another study outcome revealed an improvement of alveolar bone healing in diabetic rats [259], indicating that interfering with NLRP3 activation might be a potential therapy regarding alveolar bone loss.

Osteoarthritis (OA) is an age-related inflammatory process of the joints and can affect jaw joints, too [260]. It is characterized by the proliferation of the subchondral bone and the degeneration of articular cartilage [261]. As inflammation is the basis of OA, NLRP3 [262], proinflammatory cytokines such as IL-1β or IL-18, and ROS [263] are related to the development and progression of OA. Chen et al. [260] demonstrated that inhibition of Nrf2 expression and, further, its antipyroptosis effects, upregulate NLRP3 activation in vitro, suggesting that OA therapies targeting the Nrf2/*HO-1* signal pathway might be a promising strategy.

Besides inflammation and subsequent bone loss due to an unbalanced NLRP3 activation, and further, overexpression, interestingly, NLRP3 expressed at the physiological level may have positive regulatory effects on bone homeostasis at early ages. Detzen et al. [264] showed that NLRP3-depleted mice have a shorter/smaller habitus, due to affected long-bone growth and malfunction in osteoblast metabolism, compared to wild-type mice with unaffected NLRP3 function. Attention should be paid to the fact that this impaired skeletal development was transitory, as bone homeostasis returns to wild-type-level with age. Consequently, activation of inflammasomes might be not only a promotor of inflammation but also an outcome due to inflammatory bone loss, suggesting a positive feedback mechanism of inflammatory bone loss.

## 8. Medication-Related Osteonecrosis of the Jaw

Medication-related osteonecrosis of the jaw (MRONJ) represents a potentially severe side effect of antiresorptive (e.g., bisphosphonates, denosumab) and antiangiogenic therapies in the treatment of osteolytic processes or osteoporotic conditions. MRONJ was first described in 2003 as osteonecrosis of the jaw in patients receiving bisphosphonate therapy [265]. Bisphosphonates cause apoptosis of osteoblasts and inhibition of osteoclasts, which may lead to bone loss in the jaw [266], inter alia, due to increased inflammation [251]. In addition to osseous manifestations, loss of oral soft tissue with consequent non-healing necrotic bone [267] persisting for longer than eight weeks is part of the definition of the disease, established by the American Association of Oral and Maxillofacial Surgeons [268].

Recent studies showed that the presence of bacterial LPS during bisphosphonate therapy can induce osteonecrosis in rats, which may indicate a possible association between inflammatory pathways triggered by anaerobic bacteria and bone necrosis [269,270,271]. Anaerobic bacterial species are mainly found in bone lesions of MRONJ, suggesting that periodontal infection and inflammation support osteonecrosis, when combined with antiresorptive therapies. Vice versa, the presence of *P. gingivalis* was found to be more frequent in patients treated with bisphosphonates, indicating that antiresorptive therapies offer an ideal environment for periodontopathogenic bacteria [272].

However, exact mechanisms of MRONJ pathogenesis and related inflammatory signaling pathways still remain unclear. In this context, inflammatory processes with consequently higher levels of proinflammatory cytokines, such as IL-1β, IL-8, or TNFα, and pyroptosis of human gingival fibroblasts during bisphosphonate therapy were associated with bisphosphonate-related osteonecrosis of the jaw (BRONJ) [129,267]. It is demonstrated that bad oral hygiene and the presence of periodontopathogenic bacteria is associated with increased incidence of BRONJ [273]. In line with previous studies on NLRP3, reporting a clear relationship between the expression of the NLRP3 inflammasome and inflammatory diseases (e.g., PD), Kaneko et al. [274] also demonstrated that bisphosphonates upregulate M1-like macrophage differentiation and enhanced level of IL-1β via the NLRP3 inflammasome-dependent pathway. Several components in bisphosphonates (i.e., zoledronic acid) are known to upregulate secretion of IL-1β in murine macrophages with diabetes mellitus by activating NLRP3. Concurrent exposure to bacterial LPS increased this effect. In contrast, pharmacological NLRP3 inhibitors are demonstrated to play a role in suppressing osteonecrosis of the jaw in mice and may improve oral wound healing [129].

Lee et al. [275] demonstrated that pamidronate (i.e., a bisphosphonate) upregulates suppression of NF-κB signaling proteins, such as Nrf2. As NF-κB signaling was reduced due to pamidronate, cells showed less reaction to ROS.

In consequence, these findings suggest that osteonecrosis of the jaw during treatment with antiresorptive drugs might be regulated by the activation of the NLRP3 inflammasome signaling pathway. However, the actual role of NLRP3 or other inflammasomes in the pathogenesis of MRONJ is still unclear. Further studies are needed to point out possible relationships between osteonecrosis of the jaw due to antiresorptive therapies and inadequate activity of inflammasomes.

## 9. Calculus

Based on bad oral hygiene, oral bacterial biofilm persists on the teeth, and further, mineralizes when calcium phosphate salts precipitate in the intermicrobial matrix. Thus, dental calculus, i.e., mineralized dental plaque, occurs supra- and subgingivally, with a nonmineralized bacterial biofilm on it [276]. Dental calculus is responsible for irritation and subsequent inflammation of the gingiva [277], as it acts as a plaque-retention factor, suggesting a pathogenic potential.

Previous studies demonstrated a strong relationship between subgingival calculus and periodontal inflammation [278,279,280]. Therefore, scaling and tooth root debridement for removal of calculus is the therapy of choice regarding PD [281], and procedures with ultrasound systems for comfortable patient therapy are more popular [282].

Raudales et al. [283] showed that dental calculus induced IL-1β secretion in human polymorphonuclear leukocytes, human peripheral blood mononuclear cells, and in macrophages from wild-type mice, although, IL-1β production was inhibited in NLRP3-deficient mice. In conclusion, this study determined that, in mice and in humans, dental calculus, and partially, its crystalline structure is responsible for IL-1β formation through the activation of NLRP3.

It is already known that human epithelial cells, as the first line of the host’s defense, express NLRP3 inflammasome components [104]. Furthermore, it was demonstrated that cell death of epithelial cells is mainly induced by the inorganic component of dental calculus, which, in consequence, affects epithelial barrier functions of this cell line. Moreover, an involvement of NLRP3 inflammasome activation was indicated [284].

Cleaning the tooth root surface of periodontopathogenic bacteria and calculus remains the ultimate solution for PD prevention. Qiu et al. [285] suggested differences in the NLRP3 inflammasome activation, due to various treatments of the tooth root surface, i.e., ultrasonic scaling, hand scaling, sandblasting, or a combination. It could be concluded that there is no significant difference in the expression of NLRP3 inflammasome, and further, IL-1β secretion in human gingival fibroblasts among the different mechanical treatments leading to varying tooth root biological interfaces.

Until now, there had been no studies that examined the potential relationship between Nrf2 and dental calculus. Possible connections could be hypothesized, paying attention to the fact that, on the one hand, Nrf2 aggravates atherosclerosis. Cholesterol crystals accumulate in atherosclerotic plaques triggered Nrf2 and NLRP3 inflammasome activation, leading to IL-1 production in mice [34]. As Nrf2 is activated by cholesterol, Nrf2 is shown to be a positive regulator of the NLRP3 inflammasome.

On the other hand, Liu et al. [286] established a link between Nrf2 and intrarenal calcium oxalate crystals, suggesting that an inhibition of further inflammatory injuries to renal tubular epithelial cells is possible via the Nrf2 pathway in vivo and in vitro.

As Nrf2 is at least partly contributing to some inflammatory conditions due to crystalline structures [34,286], one might suggest that this transcription factor is involved in inflammatory responses of the gingiva due to dental calculus. Confirming studies remain missing.

## 10. Conclusions

Our review provides insight into the mechanisms and functions of the NLRP3 inflammasome and the Nrf2 transcription factor, related to the field of dental health care (Table 1). Comprehension of the oral microbiome, first in health, then in disease, is fundamental. It is of great significance to understand the contribution of several proteins, e.g., inflammasomes to diseases, and their crosstalk to signaling pathways, e.g., Nrf2, which may help investigate new therapy strategies, customized for each patient.

Inflammasomes are double-edged swords, on the one hand, as moderate inflammation has protective potential for eliminating invading pathogens, and on the other hand, uncontrolled and overexpressed inflammation leads to pulp tissue and bone necrosis.

Apart from that, Nrf2, with its antioxidant and anti-inflammatory properties, is a major regulator of other cytoprotective genes [35,36], protecting against tissue damaging.

With special regard to inflammatory oral events, there is a lack of standard guidelines in the field of dentistry in contrast to human medicine, uncovering the need of evidence-based dentistry. For inflammatory diseases (e.g., PD), a complete understanding of inflammatory signaling processes and the subsequent activity of immune cells, is elementary to modulating disease progression. Further, fundamental research on the regulation of inflammasomes may be combined with versatile drugs, may lead to patient-individualized treatments, and to specific directives for the dentist. Future studies should shed light on the possible systemic impact of initial oral inhibition of the NLRP3-pathway.

## Figures and Tables

**Table 1:** Overview of diseases and symptoms with involvement of the NLRP3 inflammasome.

Disease/Symptoms	Cause/Association	Mechanism
Periodontitis	pathogenic bacteria	Activation of NLRP3 and RANKL Nrf2 suppresses subscription of NLRP3-associated genes
Colorectal cancer	*P. gingivalis*	Activation of NLRP3
	*F. nucleatum*	Metastasis by TLR4/Keap1/Nrf2 axis
Periapical periodontis	*E. faecalis*	Lipoteichoic acid stimulating NF-κB, resulting in a decrease in osteoblasts
Bone destruction	*P. gingivalis*	Outer membrane vesicles, resulting in an increase in osteoclasts Lipopolysaccharide stimulating IL-1β
	*F. nucleatum*	via NF-κB, resulting in an increase in osteoclasts
	Calculus	Stimulating osteoclasts
Pyroptosis	*C. albicans*	Affecting macrophages and
	*P. gingivalis*	Affecting dental pulp cells
	Implants	Via Ti-Ions affecting macrophages
	Periimplants	Via Ti-Ions affecting macrophages
Oral squamous cell carcinoma	*P. gingivalis*	Via cytokines
	*F. nucleatum*	Via cytokines

## Abbreviations

5-FU	5-Fluorouracil
AIM2	absent in melanoma 2
ALRs	AIM2-like receptors
ASC	apoptosis-associated speck-like protein containing CARD
ATP	adenosine triphosphate
BRONJ	Bisphosphonate-related osteonecrosis of the jaw
CASP1	caspase-1
Cdt	cytolethal distending toxin
CLRs	C-type lectin receptors
DAMPs	damage-associated molecular patterns
GSDMD	gasdermin D
HGEC	human gingival epithelial cells
HGFs	human gingival fibroblasts
HNSCC	head and neck squamous cell cancer
*HO-1*	hemeoxygenase-1
IFN	type I interferon
Keap1	Kelch-like ECH-associated protein 1
LPS	lipopolysaccharide
LRR	leucine-rich repeat
LTA	lipoteichoic acid
Ltx	leukotoxin
MRONJ	medication-related osteonecrosis of the jaw
NF-κB	nuclear factor kappa B
NLRs	Nod-like receptors
NLRP3	NACHT domain-, leucin-rich repeat-, and pyrin domain (PYD)-containing protein 3
NOD	nucleotide-binding oligomerization domain
*NQO1*	NAD(P)H quinone oxidoreductase-1
Nrf2	nuclear factor E2-related factor or nuclear factor (erythroid-derived 2)-like 2
OA	osteoarthritis
OMVs	outer membrane vesicles
OSCC	oral squamous cell carcinoma
PAMPS	Pathogen-associated molecular patterns
PD	periodontitis
proCASP1	pro-caspase-1
PRRs	pattern-recognition receptors
PTMs	post-translational modifications
PYD	pyrin domain
RANKL	receptor activator of nuclear factor kappa-B ligand
RLRs	RIG-I-like receptors
TLRs	Toll-like receptors
VD3	1,25-dihydroxyvitamin D3
